# Redefining the N-Terminal Regulatory Region of the Ca^2+^/H^+^ Antiporter CAX1 in Tomato

**DOI:** 10.3389/fpls.2022.938839

**Published:** 2022-07-11

**Authors:** Beibei Han, Yuxin Tai, Shuping Li, Junmei Shi, Xueqing Wu, Tayebeh Kakeshpour, Jianfeng Weng, Xianguo Cheng, Sunghun Park, Qingyu Wu

**Affiliations:** ^1^Institute of Agricultural Resources and Regional Planning, Chinese Academy of Agricultural Sciences, Beijing, China; ^2^Institute of Crop Sciences, Chinese Academy of Agricultural Sciences, Beijing, China; ^3^College of Resources and Environmental Sciences, China Agricultural University, Beijing, China; ^4^College of Land and Environment, Shenyang Agricultural University, Shenyang, China; ^5^Department of Horticulture and Natural Resources, Kansas State University, Manhattan, KS, United States

**Keywords:** calcium, CAX, NRR, tomato, CRISPR

## Abstract

Calcium (Ca^2+^) is an essential plant nutrient, and Ca^2+^/H^+^ exchangers (CAXs) regulate Ca^2+^ partitioning between subcellular compartments. AtCAX1 activity is inhibited by its N-terminal regulatory region (NRR), which was initially defined as the sequence between the first two methionines. However, the accuracy of this NRR definition and the NRR regulatory mechanism remain unclear. Here, using tomato SlCAX1 as a model, we redefined the NRR of CAXs and demonstrated that our new definition is also applicable to Arabidopsis AtCAX1 and AtCAX3. The N-terminal-truncated SlCAX1 (SlCAX1^Δ39^) but not the full-length SlCAX1 was active in yeast, similar to Arabidopsis AtCAX1. Characterization of *slcax1* mutants generated by CRISPR-Cas9 confirmed the calcium transport ability of SlCAX1. Sequence alignment between SlCAX1, AtCAX1, AtCAX3, and the *Bacillus subtilis* Ca^2+^/H^+^ antiporter protein YfkE revealed that SlCAX1 does not have the 2nd methionine and YfkE does not have any amino acid residues in front of the first transmembrane domain. Truncating the amino acid residues up to the first transmembrane of SlCAX1 (SlCAX1^Δ66^) further increased its activity. The same truncation had a similar effect on Arabidopsis AtCAX1 and AtCAX3. Expression of full-length SlCAX1 and SlCAX1^Δ66^ in tomato plants confirmed the results. Our results suggest that SlCAX1 is critical for Ca^2+^ homeostasis and all the amino acid residues in front of the first transmembrane domain inhibit the activity of CAXs. Our redefinition of the NRR will facilitate fine-tuning of Ca^2+^ partitioning to reduce the incidence of Ca^2+^-related physiological disorders in crops.

## Introduction

Calcium (Ca^2+^) is an essential macronutrient for plants and functions as a second messenger that is generated in response to different stimuli. Precise regulation of its distribution between different subcellular compartments is critical for normal plant growth, development, and adaptation to the environment. Most Ca^2+^ is stored in the cell wall, apoplast, vacuole, and endoplasmic reticulum and is maintained at extremely low levels in the cytosol (Dodd et al., 2010). Ca^2+^ antiporters, such as Ca^2+^/H^+^ exchangers (CAXs), control the efflux of Ca^2+^ from the cytosol. This establishes a Ca^2+^ gradient between the cytosol and other compartments and maintains Ca^2+^ partitioning in plant cells (Emery et al., 2012).

*Arabidopsis thaliana AtCAX1,* the first identified plant CAX, was cloned based on its ability to complement the hypersensitivity of a *Saccharomyces cerevisiae* mutant with defects in vacuolar Ca^2+^ transport to high concentrations of Ca^2+^ (Hirschi et al., 1996). AtCAX1 is a high-affinity, high-capacity Ca^2+^/H^+^ antiporter. Five additional *AtCAXs* have been cloned from *Arabidopsis* (Shigaki et al., 2006). CAXs from other plant species have been intensively studied and the substrate range of these CAXs extends beyond Ca^2+^ (Kamiya et al., 2006; Pittman and Hirschi, 2016; Amagaya et al., 2020).

AtCAX1 activity is tightly regulated by its N-terminal regulatory region (NRR), which is 36 amino acids long (Pittman and Hirschi, 2001). The NRR was initially identified in the original AtCAX1 antiporter used in yeast complementation experiments. This antiporter is the product of a partial-length cDNA in which the N terminus is truncated, but there is a second in-frame start codon, allowing translation initiation from the methionine at the 37th amino acid position (referred to as AtCAX1^Δ36^ hereafter; Hirschi et al., 1996; Pittman and Hirschi, 2001). Heterologous expression of *AtCAX1*^Δ36^, but not full-length AtCAX1 (*lCAX1*), complements the hypersensitivity of the yeast mutant strain to calcium, suggesting that the first 36 amino acid residues inhibit AtCAX1 activity. The region deleted in AtCAX1^Δ36^ was defined as the NRR (Pittman and Hirschi, 2001). AtCAX3 has high amino acid sequence similarity with AtCAX1, including in the NRR. Neither full-length AtCAX3 nor the 36-amino-acid N-terminal truncation of AtCAX3 (AtCAX3^Δ36^) is as active as AtCAX1^Δ36^ in yeast expression assays. However, when longer truncations are performed, AtCAX3 is able to transport Ca^2+^ in both yeast and plants (Manohar et al., 2011). Further genetic evidence suggests that AtCAX3 functions redundantly with AtCAX1 because neither *atcax1* nor *atcax3* single mutants display an obvious phenotype, whereas the growth and development of *atcax1 atcax3* double mutants are strongly impaired (Cheng et al., 2005; Hocking et al., 2017), suggesting that AtCAX3 is an active Ca^2+^ transporter though how it is activated remains unclear.

As CAXs are important for Ca^2+^ homeostasis, tremendous effort has been made to ectopically express these genes in different economically important species, such as tomatoes, potatoes, and carrots, with the aim of improving fruit quality, abiotic stress tolerance, or shelf life, or alleviating Ca^2+^-related physiological disorders (Park et al., 2004, 2005a,b, 2009; Kim et al., 2006; Han et al., 2009; Wu et al., 2011, 2012). However, ectopic expression of *AtCAX1*^Δ36^, which is constitutively active, in certain species including tobacco and tomato resulted in strong calcium disorder symptoms such as necrosis in leaf tips or blossom-end rot (Park et al., 2005a; Wu et al., 2012). In tomato, 100% of *AtCAX1*^Δ36^-expressing plants exhibited blossom-end rot due to perturbation of calcium partitioning in plant cells (Park et al., 2005a; De Freitas et al., 2011, 2012). Although bioinformatics analysis has been performed for tomato *SlCAX* genes (Amagaya et al., 2020), their functions in Ca^2+^ homeostasis and their roles in controlling calcium-related physiological disorders remain to be addressed.

Here, we characterized the tomato *SlCAX1* gene and gained insight into its regulatory mechanism of Ca^2+^ antiporters. We redefined which region constitutes the NRR and demonstrated that our new definition could also apply to *Arabidopsis* AtCAX1 and AtCAX3. These results could facilitate efforts to mitigate physiological disorders in tomato by fine-tuning Ca^2+^ homeostasis.

## Materials and Methods

### Yeast Strains and Plant Materials

Yeast strain K667 (*vcx1::hisG cnb1::LEU2 pmc1::TRP1 ade2-1 can1-100 his3-11,15 leu2-3,112 trp1-1 ura3-1*), which lacks the vacuolar Ca^2+^-ATPase (PMC1) and vacuolar Ca^2+^/H^+^ antiporter (VCX1), was provided by Dr. Kendal Hirschi (Cunningham and Fink, 1996). For metal ion treatments, tomato plants (*Solanum lycopersicum* “Rubion”) were grown on 1/2 Murashige and Skoog (MS) medium for 2 weeks and then transferred onto an equal volume of solution containing the different metal ions and incubated for 16 h. The tissues were rinsed with distilled water three times and dried on tissue paper before flash freezing with liquid nitrogen. The frozen tissues were kept at −80°C before analysis.

### RNA Extraction and RT-qPCR

RNA was isolated using the RNeasy Plant Kit (Zymo Research), according to the manufacturer’s instructions. RT-qPCR was performed to detect *SlCAX* expression. First-strand cDNA was produced using the iScript™ cDNA Synthesis kit from BIO-RAD. The first-strand cDNA of 1 μl was used to amplify *SlCAX-specific* fragments and *SlACTIN* (acc. no. TC194780) was used as a control. Specific amplification primers are shown in Supplementary Table 1.

### Plasmid Construction and Yeast Transformation

Different versions of the *SlCAX* (*SlCAX1*, *SlCAX1*^Δ39^, *SlCAX1*^Δ58^, *SlCAX1*^Δ66^, and *SlCAX1*^Δ93^) and AtCAX (*AtCAX1*^Δ36^, *AtCAX1*^Δ63^, *AtCAX3*^Δ36^, and *AtCAX3*^Δ63^) genes were amplified from the tomato and Arabidopsis cDNA libraries, respectively, and cloned into the yeast expression vector *piHGpd* (Pittman et al., 2002). The first 39 and 66 amino acids of SlCAX1 were randomly scrambled and codon optimized based on yeast codon preference and the DNA oligos were synthesized by Beijing Dahong Biotechnology. The *scrambled-39-SlCAX1* and *scrambled-66-SlCAX1* were fused with *SlCAX1*^Δ39^ and *SlCAX1*^Δ66^, respectively, and cloned into the yeast expression vector *piHGpd*. The scrambled DNA sequences were shown in Supplementary Table 2. All PCR amplifications were performed using Phusion® High-Fidelity DNA Polymerases from New England Biolabs; the restriction sites used were XbaI and SacI. Primer sequences used in this study are shown in Supplementary Table 3.

Yeast cells were transformed using the standard lithium acetate method and selected on a synthetic defined medium lacking His as described (Pittman et al., 2002). For the Ca^2+^ tolerance assays, the yeast cells were cultured on SD-His liquid medium to an OD value of 1. After series dilution, the yeast cells were cultured on solid YPD supplemented with different concentrations of CaCl_2_ and grown at 30°C for 3 days (Pittman and Hirschi, 2001; Manohar et al., 2011).

### Preparation of Transgenic Plants

*Agrobacterium*-mediated transformation was used to generate transgenic tomato plants. Different versions of *SlCAX1* were fused with 2× Sterp tag II- 3x Flag tag driven by the CaMV35S promoter and cloned into the PRI101 vector (Satoh et al., 2004; Sugio et al., 2008). Primer sequences used for cloning are shown in Supplementary Table 2. The plasmids were introduced into *Agrobacterium* strain LBA4404 following standard protocols and then transformed into tomato (*S. lycopersicum* “Micro-Tom”) following a previously described method (Cosson et al., 2006). The transgenic lines were confirmed by genomic PCR using the following primers: SlCAX1-PRI forward, 5′-CCA ACC ACG TCT TCA AAG CA-3′ and reverse, 5′-TCC TGT AGA GTG AAA GCT GTG A-3′.

### Knock-Out of *SlCAX1* Using CRISPR-Cas9

CRISPR-Cas9 was used to create *slcax1* knockouts in tomato (Micro-Tom) through *Agrobacterium*-mediated transformation. We designed two guide RNAs (gRNAs) targeting the coding region of *SlCAX1*. The sequence of Guide1 was ‘TCG TAG CCA TGG ACG AAC GG’ and that of Guide2 was ‘CTT CTA AAT GCA ACA TGT GG’. The two guides were cloned into the CRISPR-Cas9 vector *BGK012*, which was provided by Jiangsu Baige Gene. The vector map is shown in Supplementary Figure 1. The CRISPR/Cas9 construct was transferred into the *Agrobacterium* strain LBA4404 and then into tomato, as described (Li et al., 2018). The genomic regions surrounding the target sites were PCR-amplified and Sanger sequenced. All sequence information is shown in Supplementary Table 4.

### Measurement of Ca^2+^ Contents

Seeds (T2) of the *SlCAX1*, *SlCAX1*^Δ66^-expressing, *SlCAX1* CRISPR knockout, and wild-type plants were germinated on MS medium containing MS inorganic salts and 0.8% agar (Sigma), and the pH was adjusted to 5.8–6.0 with KOH. After 4 weeks, plants of similar size from each of the lines were harvested and rinsed with deionized water two to three times before drying with filter paper (Wu et al., 2011). The plants were further dried at 80°C for 48 h. For each sample, a total of 0.5 g (dry weight) tissue was digested with 10 ml nitric acid and 0.5 ml perchloric acid at 120°C for 1 h. The amount of Ca^2+^ present in the digested tissue was determined by inductively coupled plasma optical emission spectroscopy (Tang et al., 2015).

## Results

**Figure 1:**
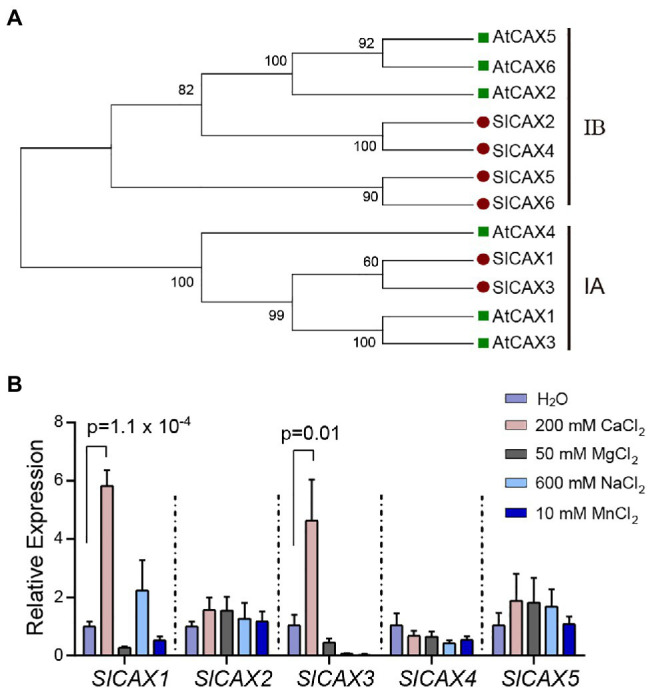
Phylogenetic and expression analysis of *SlCAXs*. **(A)** Neighbour-joining tree constructed with MEGA X software (Kumar et al., 2018). The percentages of supported bootstrap scores with 1,000 iterates are shown at the branch nodes. **(B)** Relative expression of the *SlCAX* family under different cation treatment conditions. The 14-day-old tomato seedlings were treated with different ion solutions for 16 h. Whole seedlings were collected for qPCR, and the expression of *SlCAX* genes was determined. The gene expression level under the H_2_O treatment was set as 1, and the data indicate the mean value ± SE from three biological replicates. Student’s *t*-test was used to analyse the data.

To identify SlCAX proteins from tomato, we conducted a BLAST analysis of the tomato protein database in Phytozome^1^ using the *Arabidopsis* AtCAX1 protein sequence as query. We identified six proteins with high similarity to *Arabidopsis* AtCAX1 (*Solyc09g005260*, *Solyc12g055750*, *Solyc06g006110*, *Solyc03g123790*, *Solyc07g056110*, and *Solyc12g011070*). Phylogenetic analysis using the Neighbour-Joining method identified two tomato proteins (*Solyc09g005260* and *Solyc06g006110*) in the same clade as AtCAX1, AtCAX3, and AtCAX4, which we named SlCAX1 and SlCAX3, respectively (Figure 1A). This clade, named the I-A clade, contains specialized Ca^2+^ transporters (Shigaki et al., 2006). SlCAX2, SlCAX4, SlCAX5, and SlCAX6 were grouped with AtCAX2, AtCAX5, and AtCAX6 in the I-B clade; proteins in this group transport multiple cations, including Ca^2+^, Cd^2+^, and Mn^2+^ (Figure 1A; Hirschi et al., 2000). All the tomato SlCAX proteins, except SlCAX6, were predicted to have 11 transmembrane (TM) domains (Supplementary Figure 2). Gene expression data from the Tomato eFP browser showed that *SlCAX1* and *SlCAX3* are mainly expressed in leaves, and *SlCAX2*, *SlCAX4*, and *SlCAX5* are expressed in fruits, whereas *SlCAX6* is only expressed in immature flowers (Supplementary Figure 3; Sato et al., 2012). We treated the tomato seedlings with different types of ions. Only *SlCAX1* and *SlCAX3* were dramatically induced by Ca^2+^ treatment, indicating their roles in Ca^2+^ transport (Figure 1B).

**Figure 2:**
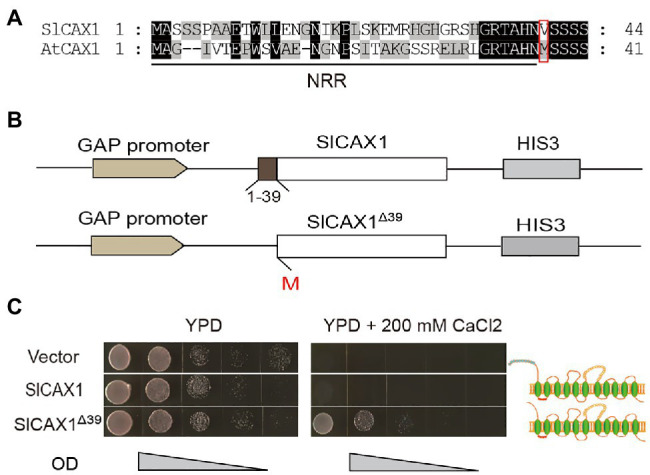
Truncating the first 39 amino acid residues of SlCAX1 increased the Ca^2+^ transport ability. **(A)** Partial amino acid sequence alignment of the N-terminal region of CAX1 from Arabidopsis and tomato. Consensus amino acid residues are boxed in black (identical) or grey (similar). Gaps introduced to maximize the alignments are denoted by hyphens. The red box indicates the 2nd methionine of AtCAX1 and the analogous valine of SlCAX1. **(B)** Schematic of *SlCAX1* and *SlCAX1*^Δ39^ expression vectors in yeast. The first 39 amino acids of SlCAX1 were truncated, and the 40^th^ amino acid, valine, was mutated to methionine, resulting in SlCAX1^Δ39^. HIS3 indicates that yeast containing this construct were able to grow on medium lacking histidine. **(C)** *SlCAX1* and *SlCAX1*^Δ39^ were expressed in the yeast strain K667. The cultures were diluted 10, 100, 1,000, and 10,000 times from OD 1.0, respectively, and an equal amount was pipetted onto yeast YPD medium with or without 200 mM CaCl_2_. The yeast cells were cultured for 3 days at 30°C.

To further verify the Ca^2+^ transport ability of SlCAX1, we heterologously expressed the encoding gene in the yeast K667 strain (*vcx1::hisG cnb1::LEU2 pmc1::TRP1 ade2-1 can1–100 his3-11,15 leu2-3,112 trp1-1 ura3-1*), which has defects in vacuolar Ca^2+^ transport (Cunningham and Fink, 1996). Expression of full-length *SlCAX1* failed to complement the hypersensitivity of the K667 strain to Ca^2+^, suggesting that, similar to AtCAX1, the full-length SlCAX1 is not active and an NRR may inhibit its activity (Pittman and Hirschi, 2001). Aligning the protein sequences of SlCAX1 and AtCAX1 revealed that the two proteins share 79% similarity and the N-terminal sequences of these two proteins are highly conserved. However, unlike AtCAX1, there is no second methionine in the N terminus of SlCAX1, and the analogous methionine at the 37th amino acid position of AtCAX1 corresponded to the valine residue at the 40th amino acid position in SlCAX1 (Figure 2A). We truncated the first 39 amino acid residues of SlCAX1 and changed valine to methionine to initiate translation. We named this N-terminal-truncated version SlCAX1^Δ39^ (Figure 2B). Complementation analysis in yeast showed that SlCAX1^Δ39^ was able to complement the hypersensitivity of the K667 strain to Ca^2+^, suggesting that SlCAX1^Δ39^ has Ca^2+^ transport ability (Figure 2C).

**Figure 3:**
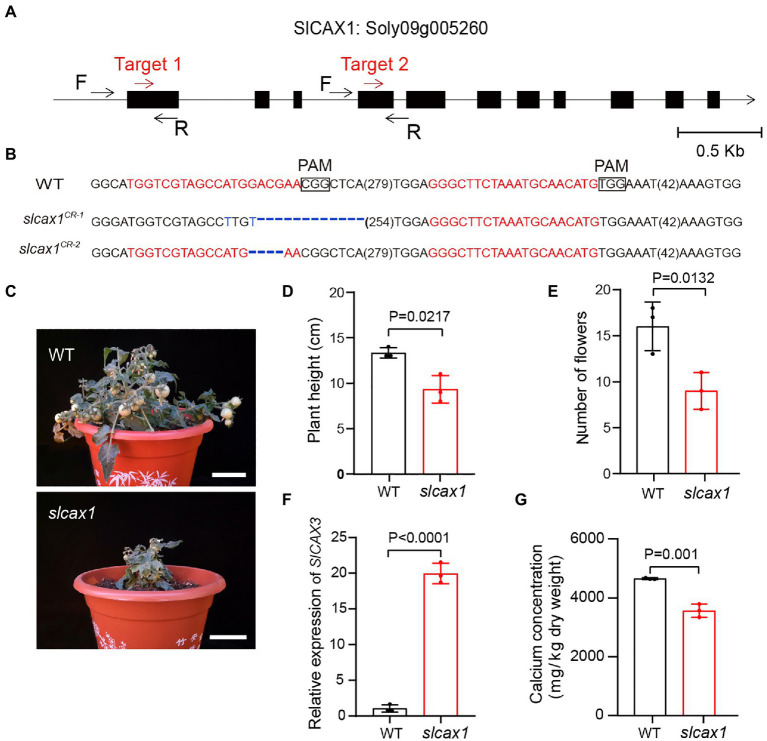
CRISPR/Cas9-engineered mutations of *SlCAX1* resulted in low Ca^2+^ concentrations in tomatoes. **(A)** The first exon of SlCAX1 was targeted by CRISPR-Cas9 using two single guide RNAs (sgRNA; target 1 and target 2; red arrows). Black arrows indicate forward (F) and reverse (R) primers used for PCR genotyping and sequencing. **(B)** Sequences of *slcax1* alleles were identified from two independent T1 transgenic plants. sgRNA targets and protospacer adjacent motif sequences are in red and bold font, respectively; deletions are indicated by blue dashes; and base mutations are shown in blue. The sequence gap length is shown in parentheses. **(C)** Phenotypes of the wild type and *slcax1* mutants. **(D)** Plant height of wild type and *slcax1* mutants grown for 2 months. **(E)** The number of flowers of wild type and *slcax1* mutants. The pictures, plant height, and flower number data were collected from 2-month-old plants. **(F)** Analysis of *SlCAX3* expression by RT-qPCR. Wild-type and *slcax1* mutant leaves were harvested 2 weeks after germination for RNA extraction from three biological replicates. **(G)** Shoot calcium content in the wild type and *slcax1* mutants. The wild type and mutants were grown on MS medium for 1 month, and then the shoots were harvested for calcium content measurement (*n* = 3). The Student’s *t*-test was used to analyze the data for **(D-G)**. Error bars represent standard error of the mean.

To confirm the Ca^2+^ transport activity of SlCAX1, we used CRISPR-Cas9 to knock out this gene. We obtained different deletions, including 37-bp and 4-bp deletions with premature stop codons predicted to result in null alleles (Figures 3A,B). The *slcax1* mutants were smaller than the wild-type plants but were able to complete the lifecycle (Figures 3C,D), and the plant height and number of flowers of *slcax1* mutants were significantly lower than those of wild-type (Figure 3E). Quantification of *SlCAX3* expression revealed that *SlCAX3* was upregulated in the *slcax1* mutants, supporting the idea of genetic redundancy between *SlCAX1* and *SlCAX3* (Figure 3F). Measuring the Ca^2+^ concentration of the wild type and mutants showed that mutants had significantly lower Ca^2+^ content (Figure 3G), confirming the Ca^2+^-transporting ability of SlCAX1. Together, the results demonstrate that SlCAX1 has a Ca^2+^ transport ability in both yeast and tomato.

**Figure 4:**
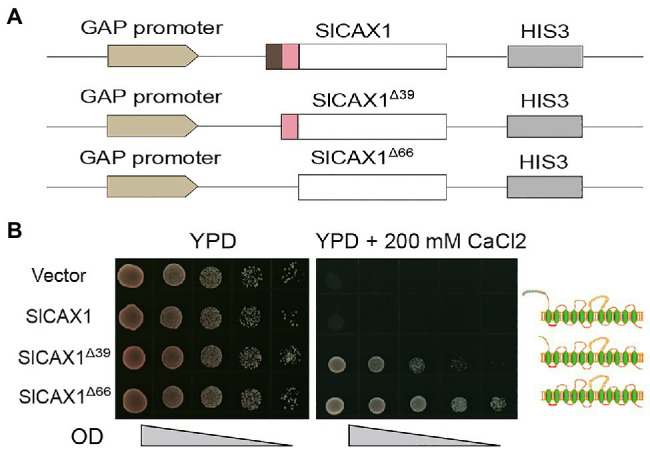
Amino acid residues before the first TM domain of SlCAX1 negatively regulate the Ca^2+^ transporting ability of the antiporter. **(A)** Schematics of the vectors used to express different truncated versions of *SlCAX1*. **(B)** *SlCAX1*, *SlCAX1*^Δ39^ and *SlCAX1*^Δ66^ were expressed in yeast strain K667. The cultures were diluted 10, 100, 1,000, and 10,000 times from OD 1.0, respectively, and an equal amount of sample was pipetted onto YPD medium with or without 200 mM CaCl_2_. The yeast cells were cultured at 30°C for 3 days.

The NRRs of *Arabidopsis* AtCAXs were initially defined by the sequence between the first and second methionine of AtCAX1 and AtCAX3; however, we found that this methionine residue is not conserved in tomato and other species (Figure 2A). Indeed, the *Bacillus subtilis* Ca^2+^/H^+^ antiporter protein YfkE, which shares high sequence similarity with *Arabidopsis* AtCAX1, does not even have any peptides in upstream of the first TM domain (Wu et al., 2013; Supplementary Figure 4). Therefore, we hypothesized that all the amino acid residues in upstream of the first TM domain of AtCAX1 negatively regulate its activity. To test this hypothesis, instead of truncating the first 39 amino acids, we truncated all 66 upstream amino acids of the first TM domain and named it SlCAX1^Δ66^ (Figure 4A). As expected, SlCAX1^Δ66^ showed higher activity than SlCAX1^Δ39^ in a yeast assay (Figure 4B), suggesting that all the amino acids in upstream of the first TM domain of SlCAX1 negatively regulate its Ca^2+^ transporting ability. To further validate our hypothesis, we made two extra truncations, SlCAX1^Δ58^ and SlCAX1^Δ93^ with the first TM domain deleted (Supplementary Figure 4). The results showed that SlCAX1^Δ66^ still displayed the highest activity in the yeast assay and contrasting to AtCAX1^Δ90^, SlCAX1^Δ93^ was not active at all, suggesting that the first TM of SlCAX1 is critical for its activity (Supplementary Figure 5). Together, our results indicate that all the amino acids in upstream of the first TM domain of SlCAX1 negatively regulate its Ca^2+^ transporting ability.

**Figure 5:**
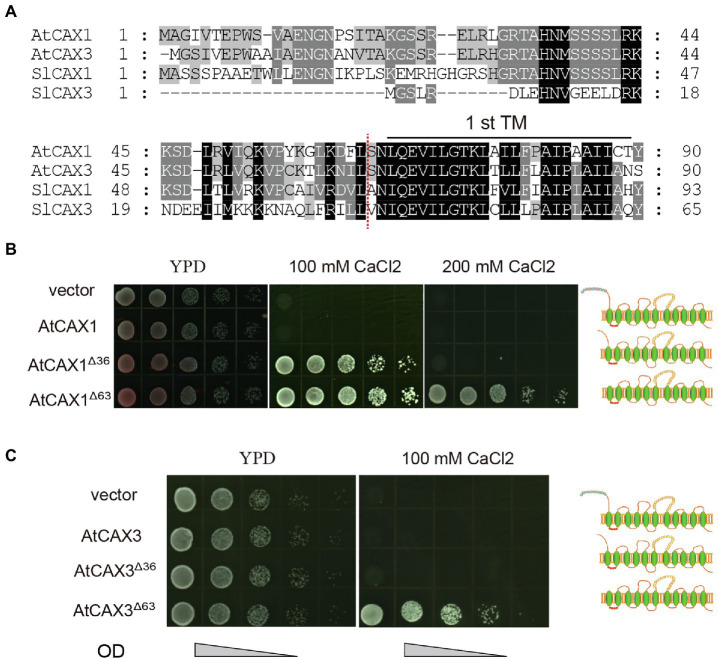
Truncating the amino acids before the first TM further increases the activity of CAX1 and CAX3. **(A)** Alignment of deduced amino acid sequences of polypeptides encoded by Arabidopsis *CAX1* and *CAX3* and tomato *SlCAX1* and *SlCAX3* (partial). Consensus amino acid residues are boxed in black (identical) or grey (similar). Gaps introduced to maximize the alignments are denoted by hyphens. The amino acids residues before the red dotted lines are deleted to form AtCAX1^Δ63^ and AtCAX3^Δ63^. **(B,C)** Truncated *AtCAX1* and *AtCAX3* were expressed in yeast mutant strain K667, respectively. The cultures were diluted 10, 100, 1,000, and 10,000 times from OD 1.0, respectively, and an equal amount of sample was pipetted onto yeast YPD medium with or without CaCl_2_. The yeast cells were cultured at 30°C for 3 days.

To determine if this is also applicable to other CAXs, such as *Arabidopsis* AtCAX1 and AtCAX3, we tested the Ca^2+^ transport activity of variants with different truncations in the K667 strain. Alignment of the protein sequences of SlCAX1, SlCAX3, AtCAX1, and AtCAX3 revealed that the sequences in upstream of the first TM domain were conserved between AtCAX1, AtCAX3 and SlCAX1, but not SlCAX3 (Figure 5A). Therefore, we truncated the first 63 amino acid residues before the first TM domains of AtCAX1 and AtCAX3 to create AtCAX1^Δ63^ and AtCAX3^Δ63^, respectively, and evaluated whether this truncation increased the activity of these proteins. Similar to SlCAX1^Δ66^, both AtCAX1^Δ63^ and AtCAX3^Δ63^ showed higher activity than AtCAX1^Δ36^ and AtCAX3^Δ36^, respectively (Figures 5B,C). The difference was particularly significant for AtCAX3 because, as indicated above, truncating the initial NRR domain of AtCAX3 does not activate this antiporter (Shigaki and Hirschi, 2000). Together, our results suggest that CAXs can be completely activated after truncating all of the amino acid residues before the first TM domain, thus our results redefine the NRR of CAXs.

**Figure 6:**
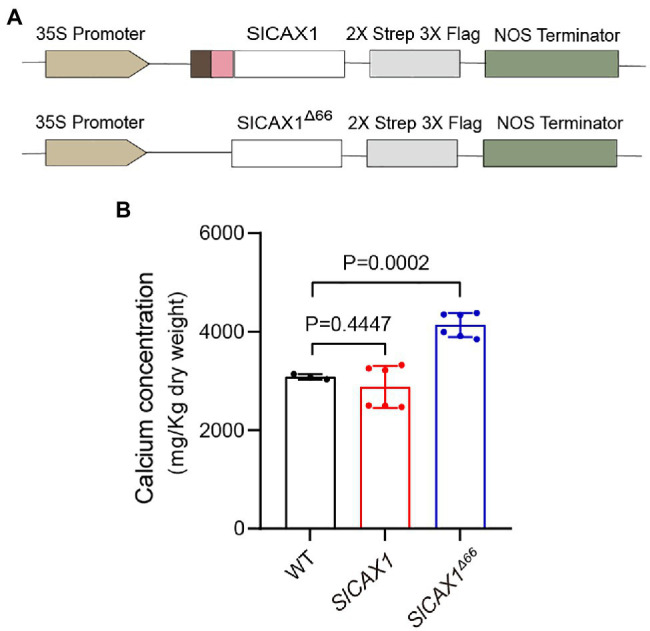
Overexpressing *SlCAX1*^Δ66^ in tomato increased the calcium content. **(A)** Schematics of vectors used to express different truncated versions of *SlCAX1* in tomato. **(B)** Calcium content of different lines. A Student’s *t*-test was used to analyze the data. Error bars represent standard error of the mean of six biological replicates.

To confirm that SlCAX1^Δ66^ has enhanced Ca^2+^ transport ability *in planta*, we expressed *SlCAX1* and *SlCAX1*^Δ66^ driven by the CaMV35S promoter in tomato plants and quantified the Ca^2+^ content (Figure 6A). We confirmed the expression using PCR (Supplementary Figure 6). Overexpression of full-length *SlCAX1* did not enhance plant Ca^2+^ content, consistent with its non-active nature. By contrast, overexpression of *SlCAX1*^Δ66^ significantly increased plant Ca^2+^ content, indicating that truncating the amino acid residues upstream of the first TM domain can activate SlCAX1 *in vivo* (Figure 6B). Together, the results from the yeast assays and the *in planta* data demonstrate that the region constituting the NRR of CAXs should be re-defined.

## Discussion

In this study, we confirmed the Ca^2+^ transporting ability of SlCAX1 using knockout mutants generated by CRISPR-Cas9 and redefined the region constituting the NRR of CAX proteins. Initially, the NRR was defined as the region between the first and second methionine of the antiporter, because both *Arabidopsis* AtCAX1 and AtCAX3 have a second methionine at the 36th amino acid position, and truncating this region increased the activity of AtCAX1 (Pittman and Hirschi, 2001). However, amino acid sequence alignment showed that the SlCAX from tomato do not have a second in-frame start codon, and some CAXs do not even have any amino acid residues in front of the first TM domain, promoting us to re-evaluate the NRR region. By testing different variants of CAXs, we established that the entire region upstream of the first TM domain inhibits CAX activity. The work by Manohar et al. also revealed that AtCAX3 with N-terminal truncation of 57 amino acids and more has the higher activity than the full length and 36-amino-acid truncation of AtCAX3 (AtCAX3^Δ36^; Manohar et al., 2011). However, how this region inhibits CAX activity remains elusive. Previous studies showed that AtCAX1 and AtCAX3 interact with each other (Cheng et al., 2005; Hocking et al., 2017), and structural analyses of the Ca^2+^ antiporter YfkE and ScVCX1 suggest that CAXs form oligomer (Waight et al., 2013; Wu et al., 2013). Therefore, it is possible that plant CAXs also form oligomeric complex. Interestingly, NRR has a high probability to consist of an intrinsically disordered region (IDR), as predicted by IUPRED3,^2^ and scrambling of the first 39 or 66 amino acids of SlCAX1 did not fully unleash its activity (Supplementary Figure 7), suggesting that the sequence of NRR does not need stringently specific to be functional. We speculate that the NRR of CAX consisting of IDR may fold back and loosely binds to its own or other CAXs’ Ca^2+^ binding sites in the complex to inhibit the Ca^2+^ transport ability. The NRR of AtCAX1 can also be phosphorylated by CBL-INTERACTING PROTEIN KINASE24 (CIPK24), and AtCAX1 is activated upon the phosphorylation (Cheng et al., 2004), indicating that activation of the CAX complex relies on other proteins. The low complexity region in the NRR consists of four serine amino acids. It is possible that phosphorylation of NRR at these amino acids could affect the interaction of NRR with the Ca^2+^ binding sites of CAX proteins and also affects the CAX activity by increasing the accessibility to Ca^2+^. However, a detailed mechanism will not be elucidated until the structure of the CAX complex is determined. Given recent advances in the cryo-EM technique, solving the structure of this membrane complex is feasible. A complete understanding of the regulatory mechanism of plant CAX proteins will benefit efforts to improve Ca^2+^ use efficiency by crop plants.

*slcax1* mutants can still complete the lifecycle, and this may be because *SlCAX1* and *SlCAX3* are genetically redundant. Similarly, neither the *Arabidopsis atcax1* nor *atcax3* single mutants showed strong growth defects, but the growth of *atcax1 atcax3* double mutants was severely hindered (Hocking et al., 2017). Our RT-qPCR results revealed that upregulation of *SlCAX3* in *slcax1* mutants allowed for functional compensation, indicating the genetic robustness of the Ca^2+^ homeostasis pathway. Genetic robustness is also common for other biological processes, such as regulation of shoot meristem development. For instance, disruption of tomato *SlCLAVATA3* resulted in upregulation of its paralogous gene *CLAVATA3/EMBRYO SURROUNDING REGION-RELATED* (*SlCLE9)*, and double mutants show synergistic phenotypes (Rodriguez-Leal et al., 2019). Further characterization of *slcax1 slcax3* double mutants would help dissect the functions of *SlCAX1* and *SlCAX3*.

Here, we used tomato, an economically important crop, as a model and determined the regulatory mechanism of CAX proteins. This study will facilitate the manipulation of Ca^2+^ antiporters in efforts to fine-tune Ca^2+^ homeostasis and thereby reduce the incidence of Ca^2+^-related physiological disorders and improve the fruit quality of crops.

## Data Availability Statement

The original contributions presented in the study are included in the article/Supplementary Material, further inquiries can be directed to the corresponding author.

## Author Contributions

BH, YT, SL, JS, XW, and TK performed the experiments. BH, YT, and QW designed research. JW, XC, SP, and QW wrote the paper. All authors contributed to the article and approved the submitted version.

## Funding

This work was supported by the National Key Research and Development Program of China (2021YFF1000400), National Natural Science Foundation of China (32171925 and U21A20210), and Innovation Program of Chinese Academy of Agricultural Sciences (GJ2022-14-5 and CAAS-ZDRW202004). The open access publication fees will be from Innovation Program of Chinese Academy of Agricultural Sciences (GJ2022-14-5).

## Conflict of Interest

The authors declare that the research was conducted in the absence of any commercial or financial relationships that could be construed as a potential conflict of interest.

## Publisher’s Note

All claims expressed in this article are solely those of the authors and do not necessarily represent those of their affiliated organizations, or those of the publisher, the editors and the reviewers. Any product that may be evaluated in this article, or claim that may be made by its manufacturer, is not guaranteed or endorsed by the publisher.

## Footnotes

^1^ https://phytozome-next.jgi.doe.gov

^2^ https://iupred.elte.hu/help_new
