# Supplementary material for: Redefining the N-Terminal Regulatory Region of the Ca2+/H+ Antiporter CAX1 in Tomato
Source: Front Plant Sci. 2022 Jul 11;13:938839. doi: 10.3389/fpls.2022.938839 (PMC9310016; doi:10.3389/fpls.2022.938839)
Supplement: Supplementary file 1 [file Data_Sheet_1.docx]

**Redefining the N-terminal regulatory region of the Ca^2+^/H^+^ antiporter CAX1 in tomato**

Beibei Han^1,6^, Yuxin Tai^2,6^, Shuping Li^1,3,6^, Junmei Shi^4^, Xueqing Wu^1^, Tayebeh Kakeshpour^5^, Jianfeng Weng^2^, Xianguo Cheng^1^, Sunghun Park^5^, Qingyu Wu^1*^


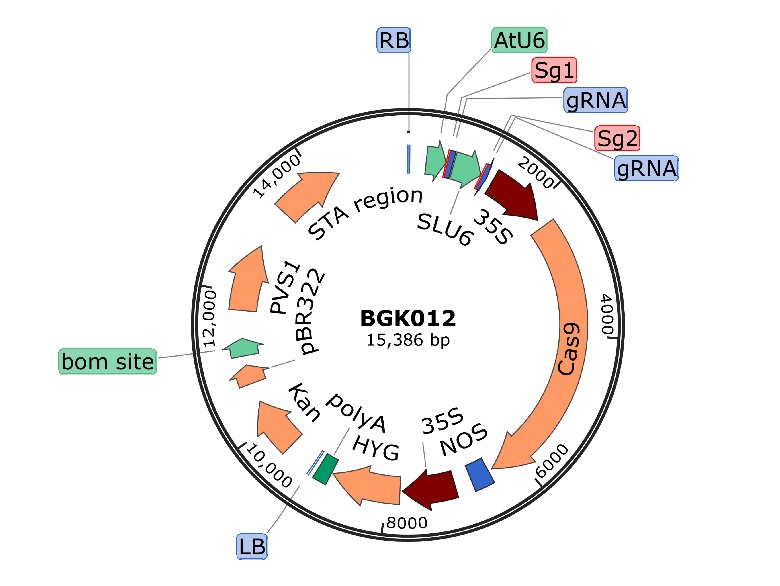


**Supplementary Figure 1.** Plasmid map of BGK012. Cas9 was driven by the *35CaMV* promoter. The selection marker genes provided kanamycin resistance in bacteria and hygromycin resistance in plants.


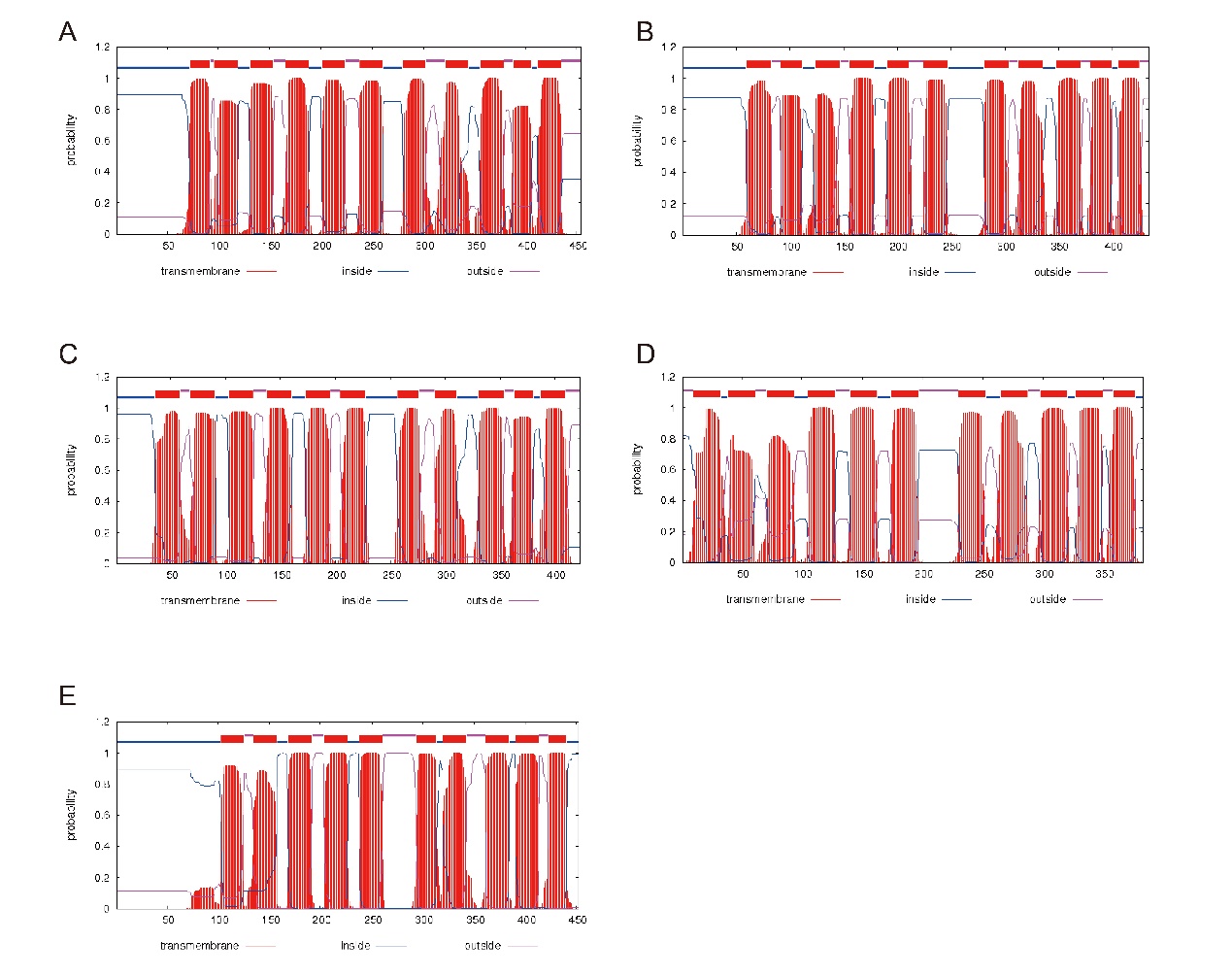


**Supplementary Figure 2**. Transmembrane structure prediction of SlCAXs. (A) *SlCAX1_Solyc09g005260*, (B) *SlCAX2_Solyc12g055750*, (C) *SlCAX3_Solyc06g006110*, (D) *SlCAX4_Solyc03g123790*, and (E) *SlCAX5_Solyc07g056110*. The probability of transmembrane locations predicted by the TMHMM algorithm is indicated by red vertical bars. The blue and magenta lines indicate inside (cytosol) and outside (vacuolar lumen) locations, respectively.


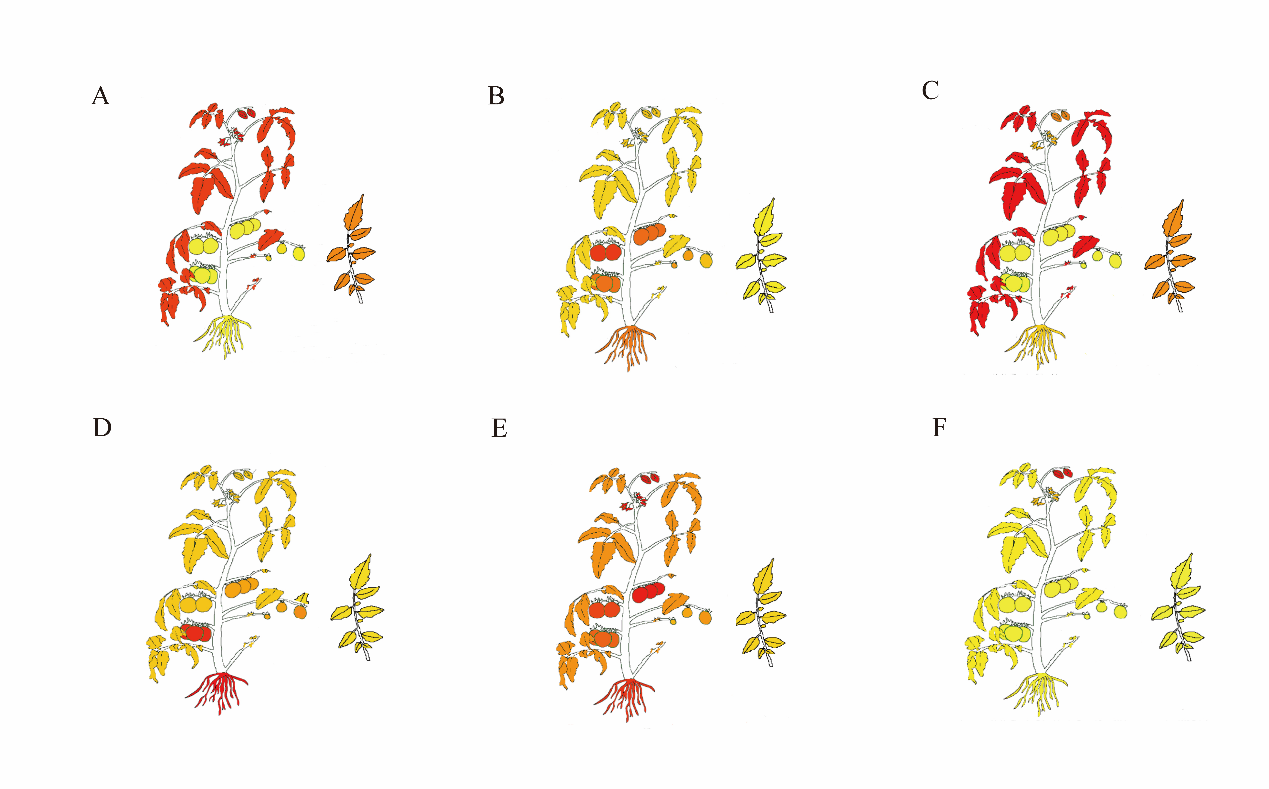


**Supplementary Figure 3.** Expression of *SlCAXs* in tomato. (A) *SlCAX1_Solyc09g005260*, (B) *SlCAX2_Solyc12g055750*, (C) *SlCAX3_Solyc06g006110*, (D) *SlCAX4_Solyc03g123790*, (E) *SlCAX5_Solyc07g056110*, and (F) *SlCAX6_Solyc12g011070*. The online tool Tomato eFP Browser was used to predict and analyse the expression of members of the *CAX* family in tomato. The colour depth represents the level of gene expression, red indicates high expression and yellow indicates low expression.

**
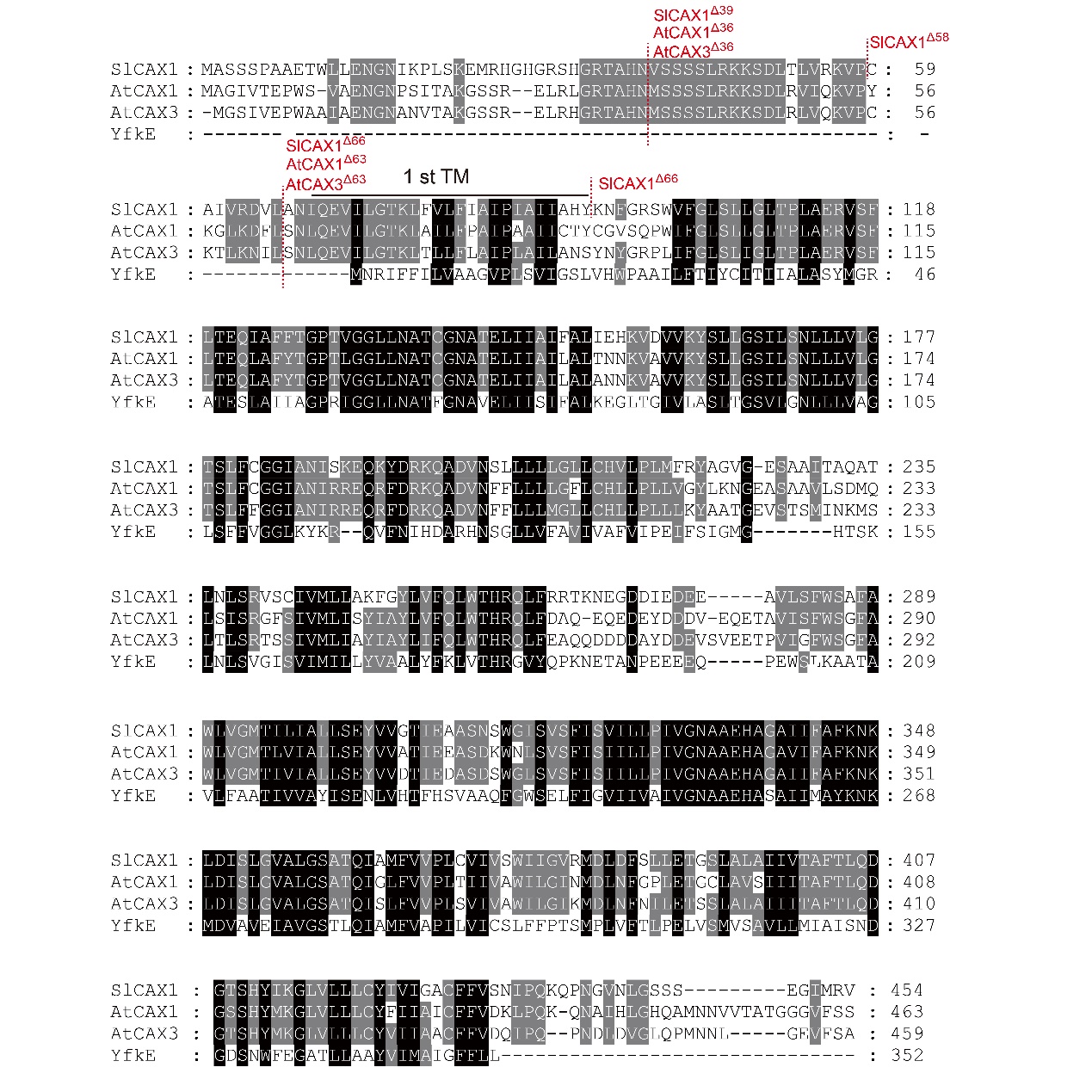
**

**Supplementary Figure 4**. Alignment of deduced amino acid sequences of polypeptides encoded by tomato *SlCAX1* and Arabidopsis *CAX1,3* and *Bacillus subtilis* *YfkE*. Consensus amino acid residues are boxed in black (identical) or grey (similar). Gaps introduced to maximize the alignments are denoted by hyphens. The amino acids residues before the red dotted lines are deleted to form SlCAX1^Δ66^, AtCAX1^Δ63^ and AtCAX3^Δ63^.


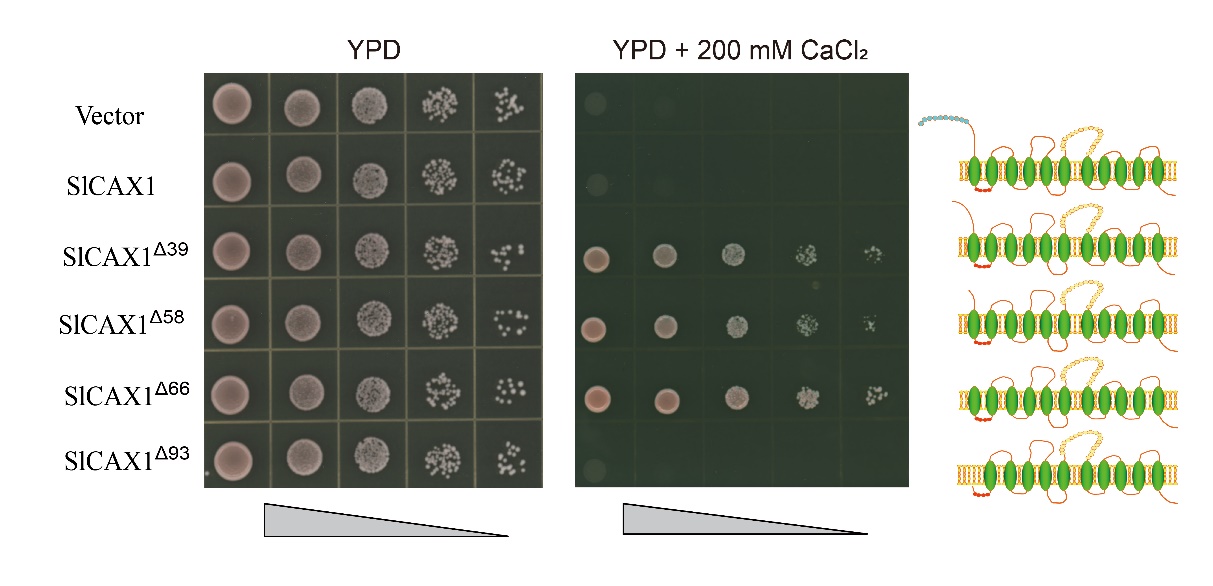


**Supplementary Figure 5**. Suppression of Ca^2+^ sensitivity of the yeast strain K667 by SlCAX1 deletion constructs. *SlCAX1*, *SlCAX1*^Δ39^, *SlCAX1*^Δ66^, *SlCAX1*^Δ58^ and *SlCAX1*^Δ93^ were expressed in yeast strain K667. The cultures were diluted 10, 100, 1000, and 10000 times from OD 1.0, respectively, and an equal amount of sample was pipetted onto YPD medium with or without 200 mM CaCl_2_. The yeast cells were cultured at 30 °C for 3 d.

**
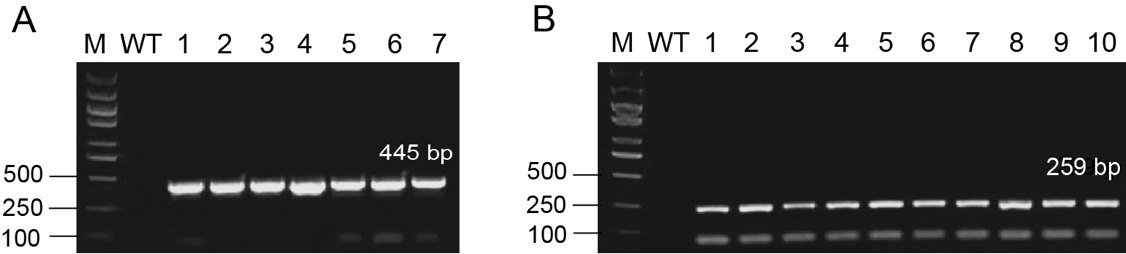
**

**Supplementary Figure 6**. Confirmation of the *SlCAX1* and *SlCAX1*^Δ66^ transgenes. Both *SlCAX1*- and *SlCAX1*^Δ66^-expression lines were confirmed by PCR using genomic DNA as templates.

**
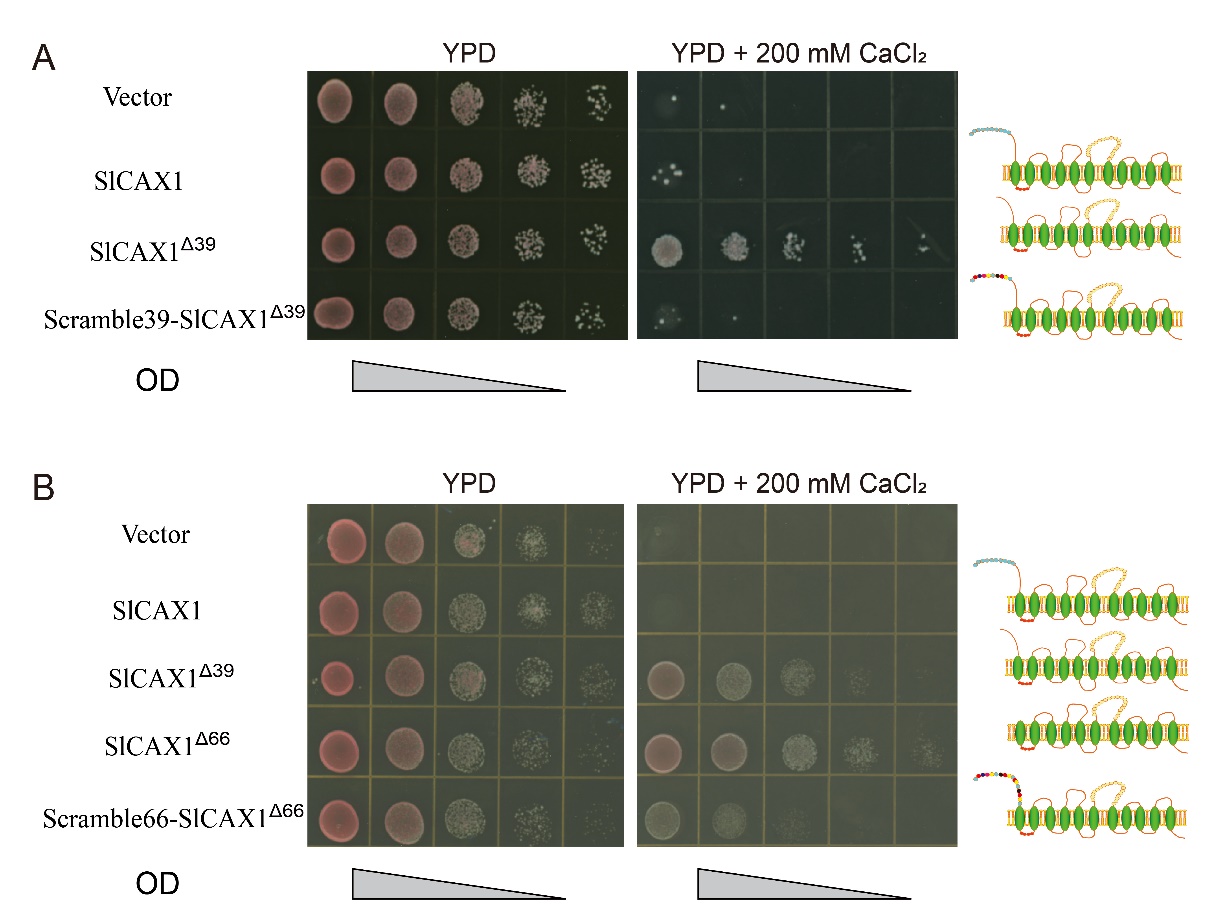
**

**Supplementary Figure 7**. Scrambling the amino acid residues before the first TM domain of SlCAX1. (A) *SlCAX1*, *SlCAX1*^Δ39^, and *Scramble39-SlCAX1*^Δ39^ were expressed in yeast strain K667. (B) *SlCAX1*, *SlCAX1*^Δ39^, *SlCAX1*^Δ66^, and *Scramble66*-*SlCAX1*^Δ66^ were expressed in yeast strain K667. The cultures were diluted 10, 100, 1000, and 10000 times from OD 1.0, respectively, and an equal amount of sample was pipetted onto YPD medium with or without 200 mM CaCl_2_. The yeast cells were cultured at 30 °C for 3 d.

**Supplementary Table 1. qRT-PCR primers used in this study**

| Primer name | Forward primer (5’-3’) | Reverse primer (5’-3’) |
| --- | --- | --- |
| qPCR-SlCAX1 | CCGACAACTTTTTCGACGCA | CCCCAAGAATTTGATGCTGCC |
| qPCR-SlCAX2 | TGCTCTTGGTGCTTGGATG | AAGCCCATGACTGCCATCAA |
| qPCR-SlCAX3 | ACGTTGTCGGTACGATCGAG | GAAGCTGACCCCAATGCAAC |
| qPCR-SlCAX4 | AAGCTATTGGGTGGCTTGCT | GGACATCGGCATGTTCAACG |
| qPCR-SlCAX5 | TGATCCGGGTGGTTCAACAG | AACAGTAGGCCCATCACTGC |
| qPCR-SlACT | GAAATAGCATAAGATGGCAGCG | ATACCCACCATCACACCAGTAT |

**Supplementary Table 2. The DNA sequences encoding the scrambled 39 and 66 amino acids of SlCAX1**

| Name | Sequence |
| --- | --- |
| Scramble 39 | ATGGGTGCTTTGTTGTCTGAATGGTTGAATCATCATACTAGACATCATGGTAGAAGATCTGGTGGTGCTCCAAATACTCCAAAATCTATGTCTGCTGCTTCTAATAAAGAAATTGAA |
| Scramble 66 | ATGCCAGCTCCAATTGGAATTAAATCACGACTTGCTTTAACATCAATGATTCGAGCTACATTATCAGCTGAATCACTTTGTCTTACATCACCATCAATTGAATTATCATGGGGACATTCAACAATTCATCGACGACTTCATAATTTACTTGATCCAGGAAATAATCTTATTCTTCATTTAGATGAATCATCATTAGGA |

**Supplementary Table 3. Primers used for vector construction in this study**

| Primer name | Primer Sequence（5’-3’） |
| --- | --- |
| pHGpd-SlCAX1-F | GAACTGTCTAGAATGGCTTCATCATCACCAGCAG |
| pHGpd-SlCAX1-R | TCAGCAGAGCTCTCAAACTCTCATGATTCCTTC |
| pHGpd-SlCAX1^Δ39^-F | ACGGATCCACTAGTTCTAGAATGTCGTCTTCGTCTCTTAG |
| pHGpd-SlCAX1^Δ66^-F | ACGGATCCACTAGTTCTAGAATGGCTAATATTCAAGAGG |
| pHGpd-SlCAX1^Δ58^-F | ACGGATCCACTAGTTCTAGAATGTGTGCCATTGTTAGA |
| pHGpd-SlCAX1^Δ93^-F | ACGGATCCACTAGTTCTAGAATGAAAAACTTTGGAAGA |
| pHGpd-SlCAX1^ΔN^-R | ACTATAGGGCGAATTGGAGCTCTCAAACTCTCATGATTCC |
| pHGpd-AtCAX1-F | ACGGATCCACTAGTTCTAGAATGGCGGGAATCGTG |
| pHGpd-AtCAX1-R | ACTATAGGGCGAATTGGAGCTCTTAACCCGTTTTAACT |
| pHGpd-AtCAX1^Δ36^-F | ACGGATCCACTAGTTCTAGAATGTCTTCTTCTTCTT |
| pHGpd-AtCAX1^Δ63^-F | ACGGATCCACTAGTTCTAGAATG TCTAATCTCCAAGAA |
| pHGpd-AtCAX3-F | ACGGATCCACTAGTTCTAGAATGTCTAATCTTCAAGAA |
| pHGpd-AtCAX3-R | ACTATAGGGCGAATTGGAGCTCTTAAGCTGAGAAAA |
| pHGpd-AtCAX3^Δ36^-F | ACGGATCCACTAGTTCTAGAATGTCTTCATCGTCGC |
| pHGpd-AtCAX3^Δ63^-F | ACGGATCCACTAGTTCTAGAATGTCTAATCTTCAAGAAG |
| PRI-SlCAX1-F | ACCCCGGGGGTACC GGATCCATGTCGTCTTCGTCTC |
| PRI-SlCAX1-R | TTCCACTTCCAGATCCATCGATAACTCTCATGATTCCTT |
| PRI-SlCAX1^Δ39^-F | ACCCCGGGGGTACCGGATCCATGGTATCGTCTTCGTCTCTTAG |
| PRI-SlCAX1^Δ66^-F | ACCCCGGGGGTACCGGATCCATGGCTAATATTCAAGAGG |

**Supplementary Table 4. PCR primers for identification of *SlCAX1* editing**

| Primer name | Primer Sequence（5’-3’） |
| --- | --- |
| CR-SlCAX1-1-F | CTAACCCCATACTCATACAAG |
| CR-SlCAX1-1-R | TTAGTTCCCAAAATGACCTCT |
| CR-SlCAX1-2-F | ACAATTTAACAACAACCTCTA |
| CR-SlCAX1-2-R | TGACACAGTTGAAATATCACT |
